# Longitudinal Cardiac Evaluation of Children with Multisystem Inflammatory Syndrome (MIS-C) Following COVID-19 by Conventional and Speckle-Tracking Echocardiography

**DOI:** 10.1007/s00246-023-03375-8

**Published:** 2024-02-19

**Authors:** Andriana Anagnostopoulou, Maria-Myrto Dourdouna, Sofia Loukopoulou, Evdoxia Mpourazani, Marios Poulakis, Evangelos Karanasios, Athanasios Michos

**Affiliations:** 1 Department of Pediatric Cardiology, “Aghia Sophia” Children’s Hospital, Athens, 11527 Greece; 2 First Department of Pediatrics, Infectious Diseases and Chemotherapy Research Laboratory, Medical School, National and Kapodistrian University of Athens, “Aghia Sophia” Children’s Hospital, Athens, 11527 Greece; 3 Pediatric Intensive Care Unit, “Aghia Sophia” Children’s Hospital, Athens, 11527 Greece

**Keywords:** MIS-C, SARS-CoV-2, COVID-19, Global longitudinal strain, Speckle-tracking echocardiography, Ventricular dysfunction

## Abstract

Multisystem inflammatory syndrome in children (MIS-C), is a rare but severe, hyperinflammatory complication of COVID-19, in which cardiovascular abnormalities are frequently detected. In this prospective study, we describe the echocardiographic findings in patients with MIS-C, with the use of conventional Echocardiography and Speckle-Tracking Echocardiography (STE) with Left Ventricular (LV) Global Longitudinal Strain (GLS) analysis, in the acute and follow-up phase. In total, 25 MIS-C patients [64% females, mean (± SD) age: 8.3 (± 3.72) years] were included. In the acute phase, median (IQR) Troponin and NT-proBNP and mean heart rate, were 8.07 (14.52) pg/mL, 2875.00 (7713.00) pg/mL, and 102.87 (± 22.96) bpm, respectively. Median (IQR) LV Ejection Fraction (LVEF) was 66 (8)% and LVEF impairment was detected in 2/25 (8%) patients. On follow-up (mean time interval:9.50 ± 4.59 months), heart rate was significantly lower, with a mean value of 90.00 (± 14.56) bpm (*p*-value = 0.017). Median (IQR) LVEF was 66.00 (6.70)% (*p*-value = 0.345) and all 25 participants had normal LVEF. In 14/25 patients, additional LV-GLS analysis was performed. During the acute phase, mean LV-GLS was − 18.02 (± 4.40)%. LV-GLS was abnormal in 6/14 patients (42.9%) and among them, only one patient had reduced LVEF. On follow-up (median (IQR) time interval:6.93 (3.66) months), mean LV-GLS was -20.31 (± 1.91)% (*p*-value = 0.07) and in 1/14 patient (7.1%), the LV-GLS impairment persisted. In conclusion, in the acute and follow-up phase, we detected abnormal LV-GLS values in some patients, in the presence of normal LVEF, indicating that STE-GLS is a valuable tool for identifying subclinical myocardial injury in MIS-C.

## Introduction

Despite the milder clinical course of SARS-CoV-2 infection observed in children in comparison to adults, cases of a delayed hyperinflammatory condition, known as Multisystem Inflammatory Syndrome in children (MIS-C), occurring 2–6 weeks after acute SARS-CoV-2 infection, have been described [1–5]. This syndrome is characterized by persisted fever and multiorgan dysfunction, which are accompanied by laboratory evidence of inflammation [4, 6–8]. Although a rare sequela of SARS-CoV-2, MIS-C can lead to serious and life-threatening complications that require prolonged hospitalization and Intensive Care Unit (ICU) admission [8–10].

In MIS-C, cardiac involvement is frequent, as it has been estimated that approximately up to 80% of affected children develop manifestations from the cardiovascular system [11–13]. These manifestations include ventricular dysfunction, valvular dysfunction, pericardial effusion, arrhythmias, conduction disorders, Coronary Artery Abnormalities (CAA) and in severe cases acute heart failure and cardiogenic shock [5, 11, 14–16]. From the above, the most common cardiac finding in MIS-C, is Left Ventricular (LV) systolic myocardial dysfunction, which may range from subclinical myocardial injury to severe dysfunction [5, 11, 13, 16, 17]. Within days to weeks from the acute illness, the cardiac manifestations, that are described in this syndrome, usually resolve and conventional echocardiographic parameters like the LV Ejection Fraction (LVEF) return to normal, especially after the administration of effective treatment [14, 18, 19]. Nevertheless, it has been reported that despite preserved LVEF, cardiac strain abnormalities can be present in some patients, suggesting subclinical myocardial damage [4, 14, 19]. Thus, the actual incidence of ventricular dysfunction in patients with MIS-C, both acutely and in the long-run, might be underestimated by conventional Echocardiography [14, 15].

In this context, Speckle-Tracking Echocardiography (STE) has emerged as an advanced angle-independent echocardiographic technique, that assesses the myocardial strain and thereby the LV deformation, by tracing the speckle displacement during the cardiac circle [13, 20, 21]. In comparison with conventional Echocardiography, STE displays greater reproducibility and sensitivity in detecting subclinical and subtle LV systolic dysfunction [17, 21, 22]. In pediatric cardiology, STE has been used for strain assessment in various clinical settings, such as in acute myocarditis, myocardiopathy related to muscular dystrophy, cancer-therapy-induced cardiοtoxicity and Kawasaki Disease (KD) and may detect subclinical myocardial damage, in the presence of preserved LVEF [21, 23].

However, data regarding the cardiac effects of MIS-C post-hospital discharge and the evaluation of the mid-term and long-term cardiac complications with the use of STE, are still lacking [9, 11, 12, 18, 19]. Therefore, in the present study, we aimed to describe the cardiac findings in patients with MIS-C with the use of conventional echocardiography and STE with LV Global Longitudinal Strain (GLS) analysis in the acute setting and in a follow-up period of at least one-month post-discharge.

## Materials and Methods

### Study Design and Participants

This was a single-center prospective study conducted at the “Aghia Sophia” Children’s Hospital, Athens, Greece, which is the largest tertiary pediatric hospital in Greece and a reference center for pediatric heart disease. Children aged 0–16 years old with a diagnosis of MIS-C, who were admitted to the hospital from January 01, 2021, to September 30, 2022, were included in the study. All study participants fulfilled the Centers for Disease Control and Prevention (CDC) and/or the World Health Organization Criteria (WHO) MIS-C criteria [24, 25]. A patient was excluded from the study if he had pre-existing heart failure, congenital heart disease that required surgical intervention or any other form of severe cardiac disease.

Patient information such as demographic (age, gender) and clinical (comorbidities, heart rate, blood pressure, clinical presentation, Intensive Care Unit admission, treatments, outcome) data were obtained. Blood pressure and heart rate measurements were obtained with the use of a vital signs monitor (GE HealthCare, Chicago, IL, USA). In addition, the following laboratory data were recorded: complete blood count, inflammation markers including C-reactive protein (CRP), procalcitonin, ferritin and cardiac biomarkers, including high sensitivity (hs)-Troponin T (cut-off level = 14 pg/mL) and N-terminal prohormone of B-type Natriuretic Peptide (NT-proBNP). The cut-off level of NT-proBNP was determined according to published normal reference values per age [26]. Subclinical myocardial injury was defined as an early cardiac injury without any clinical evidence of coronary heart disease or heart failure [27].

At admission, children underwent a comprehensive echocardiographic assessment with conventional as well as strain measurements. The echocardiographic assessment was repeated at the follow-up period of at least one-month post-hospital discharge.

## Echocardiographic Examination

### Conventional Echocardiography

Transthoracic 2-Dimensional (2D) Echocardiographic examination was performed by two experienced pediatric cardiologists, using the Vivid E90 and Vivid E7 Ultrasound System (GE HealthCare, Chicago, IL, USA), according to the guidelines and standards for performing a pediatric echocardiogram of the American Society of Echocardiography [28]. The following echocardiographic parameters were recorded: aortic dimension, left atrial diameter, pulmonary artery diameter, LV diastolic diameter and LV systolic diameter. The LVEF and the Fractional Shortening (FS) were calculated by M-mode measurements. LVEF over 55% (LVEF ≥ 55%) and FS from 28% to 46% were considered normal [29]. Parameters of the coronary arteries like left coronary diameter, right coronary diameter, left circumflex and left anterior descending branch were also measured. In accordance with the American Heart Association guidelines for KD, classification of CAA was performed based on the Z-score system as following: no coronary involvement (Z-score: always < 2), dilation only (Z-score: 2 to < 2.5 or if initially < 2, an ≥ 1 decrease in Z-score during follow-up), small aneurysm (Z-score: ≥2.5 to < 5), medium aneurysm (Z-score: ≥5 to < 10 and absolute dimension < 8 mm) and large or giant aneurysm (Z-score: ≥10 or absolute dimension ≥ 8 mm) [30].

Diastolic function was assessed with the use of Pulsed Wave (PW) Doppler and Tissue Doppler Imaging (TDI). The parameters early (E) and late (A) peak mitral inflow velocity and E/A ratio, were calculated by PW Doppler [31]. Peak velocities of basal segments were assessed by TDI and subsequently the e prime (e’) in the septal and lateral aspect of the left heart and the E/e’ ratio were calculated [32]. Diastolic dysfunction was defined as an impairment of at least two out of the three following parameters: E/A, e’, E/e’ [33]. The mitral inflow E/A Doppler profile was considered abnormal if the E and A waves were fused or if the E/A ratio had a Z-score > 2.0 [33]. The e’ velocity and E/e’ ratio, either septal or lateral, were considered abnormal if their Z-score was > 2.0 [33]. Normal reference data for age were used for the calculation of Z-scores [34, 35].

### Speckle-Tracking Echocardiography (STE)

In parallel, for the measurement of peak systolic longitudinal strain, STE with LV-GLS analysis, was performed real time by the same cardiologists that performed the echocardiographic examination with conventional echocardiographic techniques, using the GE Automated Function Imaging (AFI) Software (GE Healthcare, Chicago, IL, USA). Specifically, echocardiography loops were selected from the most appropriate apical 4-, 3- and 2- Chamber (C) views [36]. Subsequently, a line was loosely traced along the endocardium of the left ventricle [36]. The software selected acoustic markers which followed the myocardial movement [36]. The contractility in the area selected was measured by automatic frame-by-frame tracking of these markers [36]. GLS and GLS rate were calculated for the entire trail of the myocardium of the left ventricle (basal, mid, and apical segments of 2 opposite walls in each view) [36]. GLS values were not included in the analysis, if more than three segments were rejected [22]. As per published literature, GLS values >-18% indicated reduced systolic function [11, 37].

### Ethical Issues

The study was conducted according to the Declaration of Helsinki. Written informed consent was obtained from the participants parents or legal guardians. The study protocol was approved by the scientific and bioethics committee of “Aghia Sophia” Children’s Hospital (No 5893).

### Statistical Analysis

Absolute and relative frequencies (%) were used to describe the qualitative variables such as demographics and categorized variables (normal vs. abnormal). Categorical variables were analyzed with Fisher’s exact test. Mean, Standard Deviation (SD), median, and interquartile range (IQR) were used for quantitative data. Continuous variables that were normally distributed, were analyzed with the *t*-test. Differences between paired samples were assessed using paired *t*-test or Wilcoxon Signed Rank test, respectively for normal and non-normal data. McNemar test was used for associations between baseline and follow-up measurements for categorized data. Correlations between continuous variables were performed with the Pearson correlation coefficient (r). The assumption of normality was checked through kurtosis and skewness. The statistical significance level was set at *p*-value ≤ 0.05. Statistical analysis was performed using SPSS version 26.0 (IBM Corp., Released 2019. IBM SPSS Statistics for Windows, Version 26.0. Armonk, NY: IBM Corp).

## Results

### Patients’ Baseline Characteristics

In the present study, a total of 25 children diagnosed with MIS-C were included. The mean (± SD) age of the children was 8.30 (± 3.72) years. Among the study participants, 16/25 (64%) were females and 9/25 (36%) were males. The baseline demographic, clinical and laboratory characteristics of the study population are presented in Table 1.

**Table 1:** Descriptive statistics of demographic and clinical characteristics, and laboratory measurements in children diagnosed with MIS-C

Demographic, Clinical and Laboratory Parameters	
Gender female*	16 (64)
Underlying Comorbidities*	3 (12)
Symptoms*	
Fever	25 (100)
Gastrointestinal Symptoms	21 (84)
Cardiovascular Involvement	20 (80)
Mucocutaneous Involvement	16 (64)
Respiratory Symptoms	8 (32)
Neurocognitive Symptoms	4 (16)
Treatment*	
IVIG	25 (100)
Corticosteroids	24 (96)
Aspirin	11 (44)
Biologic Agents (Anakinra)	6 (24)
Antibiotics	22 (88)
PICU admission*	14 (56)
Mortality*	0 (0)
Complete Blood Count in the acute phase	
Haemoglobin (g/dL)	10.78 ± 1.13
White blood cells (10^3^ cells/µL)^‡^	11.64 (7.40)
Neutrophils (10^3^ cells/µL)	10.15 ± 5.50
Lymphocytes (10^3^ cells/µL)^‡^	1.28 (1.09)
Platelets (10^3^ cells/µL)	266.68 ± 131.26
Inflammatory markers in the acute phase	
CRP (mg/L)	74.93 ± 61.68
Procalcitonin (µg/L)^‡^	1.04 (1.28)
Ferritin (µg/L)^‡^	347.00 (748.00)
Cardiac enzymes in the acute phase	
hs-Troponin T (pg/mL)^‡^	8.07 (14.52)
NT-proBNP (pg/mL)^‡^	2875.00 (7713.00)

*Notes*: Values are referred to as: mean ± standard deviation (SD), ^‡^median (interquartile range) or ^*^absolute frequencies (relative frequencies, %)

Abbreviations: MIS-C; Multisystem Inflammatory Syndrome in children after COVID-19, IVIG; Intravenous Immune Globulin, PICU; Pediatric Intensive Care Unit, CRP; C-reactive protein, hs-Troponin T; high sensitivity Troponin T, NT-proBNP; N-terminal prohormone of brain natriuretic peptide

Most of the children that participated in the study (22/25, 88%) were previously healthy (Table 1). Three children had underlying comorbidities. Specifically, one child had ventricular septal defect and a bicuspid aortic valve, another child had a history of transfusion-dependent homozygous beta-thalassemia, and a third child was diagnosed with Noonan’s syndrome without cardiac manifestations.

The most common symptom in our cohort of MIS-C patients was fever (25/25, 100%), followed by gastrointestinal symptoms (21/25, 84%). Cardiovascular involvement was also frequent, as it was observed in 20 out of 25 participants (80%). In the acute phase, the most common treatments were Intravenous Immunoglobulin (IVIG) (25/25, 100%) and corticosteroids (24/25, 96%). Most children also received antibiotics (22/25, 88%), while 11 out of 25 (44%) received aspirin and 6 out of 25 (24%) were treated with the biological agent Anakinra. Admission to the pediatric ICU was required in 14 out of 25 children (56%). No death was reordered in our cohort and all MIS-C patients recovered (Table 1).

At admission, serum concentrations of inflammatory markers were elevated in the majority of the participants. Specifically, mean CRP (± SD) was 74.93 ± 61.68 mg/L and most participants (19/25, 76%) had raised levels of CRP. The median (IQR) serum concentration of ferritin was 347.00 (748.00) µg/L and the median (IQR) Procalcitonin was 1.04 (1.28) µg/L (Table 1).

Regarding the cardiac biomarkers, at admission, median (IQR) hs-Troponin T was 8.07 (14.52) pg/mL and hs-Troponin T was elevated in 7 out of 25 participants (28%). Median (IQR) NT-proBNP was 2875.00 (7713.00) pg/mL and most of the participants (20/25, 80%) had NT-proBNP concentrations above the cut-off level (Table 1).

### Cardiological Assessment

MIS-C patients underwent cardiological evaluation at admission and at a follow-up of a mean (± SD) time interval of 9.5 (± 4.59) months (range:1.20-18.56 months). In a subset of patients (14/25), in parallel with conventional echocardiographic examination, STE-GLS analysis was performed. It should be noted that in some patients the GLS could not be calculated due to poor echocardiographic windows or differences in heart rate. Only patients that had GLS images of adequate quality at both time-points (admission and follow-up) were kept in the statistical analysis of GLS. For this specific group of patients, median (IQR) time interval of the follow-up was 6.93 (3.66) months (range:5.3–18 months). The findings of Heart rate measurement, conventional Echocardiography and STE with LV-GLS analysis are presented in Table 2.

**Table 2:** Heart rate and echocardiographic findings of Standard echocardiography and Speckle-tracking echocardiography with left ventricular global longitudinal strain analysis at admission and on the follow-up period of children diagnosed with MIS-C

Parameters	Admission		Follow-up	*p*-value
Heart Rate (bpm)		102.87 ± 22.96	90.00 ± 14.56	0.017^a^
		(n = 15)	(n = 15)	
*Standard Echocardiography*				
LVEF (%)^‡^		66.00 (8.00)	66.00 (6.70)	0.345^b^
		(n = 25)	(n = 25)	
Number of children (%)* with normal LVEF		23/25 (92.0%)	25/25 (100%)	-
		(n = 25)	(n = 25)	
SV (mL)^‡^		51.40 ± 22.58	49.20 ± 13.22	0.776^a^
		(n = 5)	(n = 5)	
FS (%)^‡^		35.50 (7.00)	36.00 (6.00)	0.601^b^
		(n = 21)	(n = 21)	
EDV (mL)^‡^		69.41 (30.00)	72.41 (25.00)	0.557^b^
		(n = 17)	(n = 17)	
ESV (mL)^‡^		23.35 (9.00)	26.29 (7.00)	0.448^b^
		(n = 17)	(n = 17)	
*Speckle-Tracking Echocardiography*				
GLS (%)		-18.02 ± 4.40	-20.31 ± 1.91	0.070^a^
		(n = 14)	(n = 14)	
Portion of children (%)* with normal GLS		8/14 (57.1%)	13/14 (92.9%)	0.063^c^

*Notes*: Values are referred to mean ± standard deviation (SD), ^‡^median (interquartile range) or ^*^absolute frequencies (relative frequencies, %). *P-*value obtained after: ^a^paired samples t-test, ^b^Wilcoxon Signed rank test, ^c^McNemar test

Abbreviations: MIS-C; Multisystem Inflammatory Syndrome in children after COVID-19, bpm; beats per minute, LVEF; Left Ventricular Ejection Fraction, SV; Stroke Volume, FS; Fractional Shortening, GLS; Global Longitudinal Strain

### Findings at Admission

At admission, the mean (± SD) heart rate was 102.87 (± 22.96) beats per minute (bpm) (n = 15) and median (IQR) systolic heart pressure was 80.00 (30.00) mmHg (n = 13). Regarding the assessment of systolic heart function with conventional 2D Echocardiography, at admission, median (IQR) LVEF was 66.00 (8)% and only in two participants (2/25, 8%) the LVEF was depressed. The mean SV (n = 5) was 51.40 (± 22.58) mL and median (IQR) FS was 35.50 (7.00)% (Table 2). FS was normal in 20 out of 21 participants (95.2%). In addition, diastolic function was also assessed with the use of conventional echocardiography in a subset of the participants (n = 6) at admission. All participants that were evaluated at admission, had normal diastolic function. Mean (± SD) A wave was 0.61 (± 0.16) m/s, mean (± SD) E/A ratio was 1.51 (± 0.39), mean (± SD) e’ lateral was 0.14 (± 0.02) m/s, mean (± SD) e’ septal was 0.12 (± 0.01) m/s and mean (± SD) Ε/e’ ratio was 6.96 (± 0.96).

Most of the patients that where assessed had to some extent valvular insufficiency. Specifically 13 out of 15 patients (86.7%) had mitral regurgitation. From them, all patients had mild mitral regurgitation, but one participant was diagnosed with moderate mitral regurgitation. Additionally, 13 out of 13 patients had mild tricuspid regurgitation. Finally, on the acute setting, only one child (1/18, 5.5%) had developed coronary artery dilation which eventually resolved on follow-up.

In parallel, in a subset of the participants (n = 14), systolic heart function was also assessed with STE with LV-GLS analysis. The demographic and clinical characteristics of this subgroup of patients are presented at Table 3. Τhe mean (± SD) age of this subgroup of patients was 9.00 (± 3.97) years. Half (n = 7) of the children were admitted to the PICU and all children in this group were treated with IVIG and steroids. At admission, mean (± SD) LV-GLS was − 18.02 (± 4.40)%. LV-GLS was abnormal (>-18) in 6/14 (42.9%) cases (Table 2). Among the six patients that had impaired LV-GLS, five patients had preserved LVEF. There was no statistically significant difference in the demographic and clinical characteristics between the patients that had impaired LV-GLS and those that had normal LV-GLS at admission (Table 3). Heart rate did not have a statistically significant correlation with LV-GLS at admission (r = 0.333, *p-*value = 0.245, n = 14).

**Table 3:** Clinical and demographic characteristics of 14 children diagnosed with MIS-C and evaluated with speckle-tracking echocardiography with Left Ventricular Global Longitudinal Strain (LV-GLS) analysis

	Children evaluated with STE-GLS (n = 14)	Impaired GLS at admission (n = 6)	Normal GLS at admission (n = 8)	*p*-value
Age (years)	9.00 ± 3.97	8.91 ± 3.59	9.07 ± 4.48	0.944^a^
Gender female*	7 (50)	2 (33.3)	5 (62.5)	0.592^b^
Underlying Comorbidities*	1 (7.1)	0 (0)	1 (12.5)	> 0.999^b^
Symptoms*				
Fever	14 (100)	6 (100)	8 (100)	-
Gastrointestinal Symptoms	12 (85.7)	5 (83.3)	7 (87.5)	> 0.999^b^
Cardiovascular Involvement	10 (71.4)	5 (83.3)	5 (62.5)	0.580^b^
Mucocutaneous Involvement	10 (71.4)	6 (100)	4 (50)	0.085^b^
Respiratory Symptoms	4 (28.6)	0 (0)	4 (50)	0.085^b^
Neurocognitive Symptoms	0 (0)	0 (0)	0 (0)	-
Treatment*				
IVIG	14 (100)	6 (100)	8 (100)	-
Corticosteroids	14 (100)	6 (100)	8 (100)	-
Aspirin	6 (42.9)	2 (33.3)	4 (50)	0.627^b^
Biologic Agents (Anakinra)	4 (28.6)	3 (50)	1 (12.5)	0.245^b^
Antibiotics	13 (92.9)	6 (100)	7 (87.5)	> 0.999^b^
PICU admission*	7 (50)	3 (50)	4 (50)	> 0.999^b^

*Notes*: Values are referred to mean ± standard deviation (SD) or ^*^absolute frequencies (relative frequencies, %). *p-*value obtained after: ^a^t-test, ^b^Fisher’s exact test

Abbreviations: MIS-C; Multisystem Inflammatory Syndrome in children after COVID-19, GLS; Global Longitudinal Strain, IVIG; Intravenous Immune Globulin, PICU; Pediatric Intensive Care Unit

### Findings during the Follow-up

During the long-term follow-up, mean heart rate was statistically significantly lower at 90.00 (± 14.56) bpm, than at admission (*p*-value = 0.017). Median (IQR) LVEF was 66.00 (6.70)%, Mean (± SD) SV was 49.20 (± 13.22) mL and median (IQR) FS was 36.00 (6.00)%. The differences in the FS and SV measurements were not statistically significant at follow-up compared to admission (Table 2). On follow-up, LVEF and FS were normal in all the participants that were measured (25/25, 100% and 21/21, 100%, respectively). Regarding the diastolic heart function, all participants that were evaluated (n = 15), had normal diastolic function at follow-up. Mean (± SD) A wave was 0.59 (± 0.15) m/s, median (IQR) E/A ratio was 1.50 (0.22), median (IQR) e’ lateral was 0.16 (0.03) m/s, mean (± SD) e’ septal was 0.13 (± 0.02) m/s and mean (± SD) Ε/e’ ratio was 5.93 (± 1.42).

As for valvular dysfunction, at follow-up 10 out of 15 (66.7%) patients were diagnosed with mild mitral regurgitation (*p*-value = 0.453). Regarding the tricuspid valve, at follow-up, 13 out of 13 patients were diagnosed with mild tricuspid regurgitation.

**Fig. 1:**
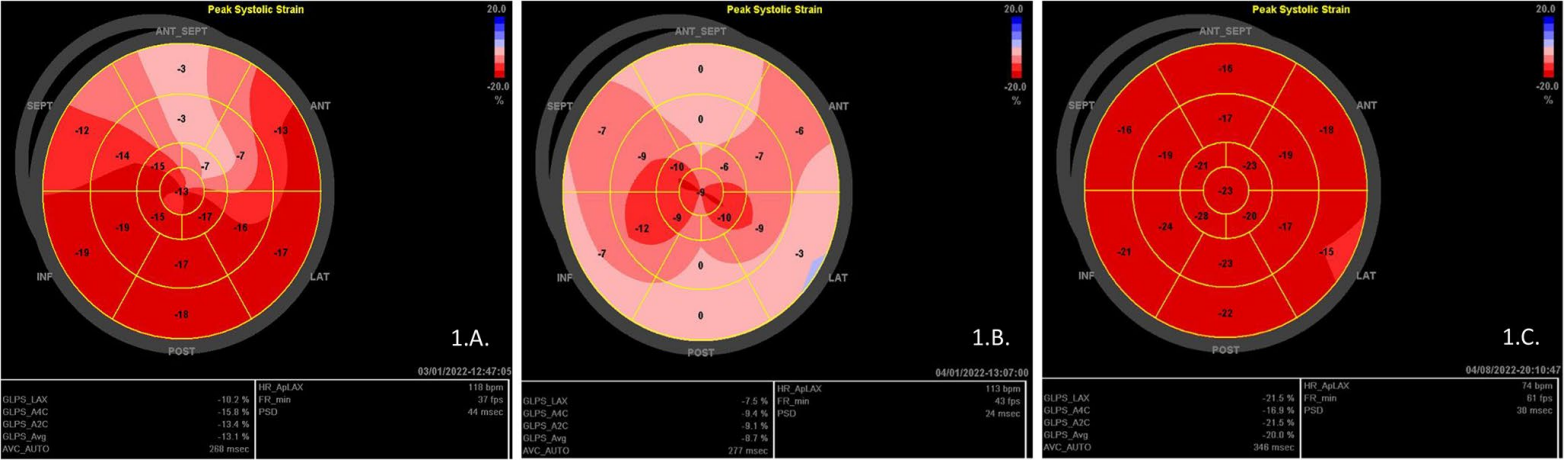
Evolvement of myocardial strain abnormalities detected on a patient diagnosed with MIS-C. At admission, while the patient had normal systolic function parameters assessed by conventional Echocardiography, strain rate was abnormal on the anterior septal segment of the left ventricle (**A**). Three days after the admission, strain measurements further deteriorated, demonstrating generalized global hypokinesis of the left ventricular wall (**B**). Seven months later, there was full resolution of the myocardial dysfunction, with normalization of the systolic and diastolic function (**C**). The bull’s eye of the strain rate was normal (**C**)

In the subset of participants (n = 14), in which LV-GLS analysis was performed, on follow up, mean (± SD) LV-GLS was − 20.31 (± 1.91)% (*p*-value = 0.07). LV-GLS returned to normal in most of the participants, that had abnormal GLS in the acute setting (Fig. 1), (*p*-value = 0.063). Only in one patient (1/14, 7.1%) LV-GLS was abnormal on follow-up, despite the presence of normal LVEF (Table 2). This child did not have any underlying disease. Heart rate did not correlate significantly with LV-GLS at follow-up (r=-0.284, *p-*value = 0.325, n = 14). The evolvement of LV-GLS impairment of a patient with MIS-C, from the acute phase until a seven-month follow-up, is presented in Fig. 1.

### Discussion

In MIS-C, cardiovascular involvement is not only one of the most prominent features, but it is also a key determinant of illness severity, management, and prognosis [12, 15]. To date, most published reports assess myocardial function in MIS-C with conventional echocardiography, while very few studies use advanced echocardiographic techniques like STE in a long-term follow-up of these patients [4, 11, 12, 18]. Therefore, in this single-center prospective study, we performed echocardiographic evaluation of children presenting with MIS-C, with conventional Echocardiography and in a subset of patients we performed additionally STE with LV-GLS analysis, at admission and in a follow-up period of a mean time interval of 9.5 months.

The demographic, clinical and laboratory characteristics of the children diagnosed with MIS-C that participated in our study, were relatively consistent with those previously described in literature, with fever, evidence of inflammation, gastrointestinal and cardiovascular involvement being the most prevalent features in our cohort [38]. Regarding specifically the cardiac involvement, our main findings included LV myocardial dysfunction and mild valvular insufficiencies.

In the present cohort, impaired LV systolic myocardial function was detected in a subset of patients, in the acute setting, with both echocardiographic methods used. Particularly, at admission, LVEF was reduced in 8% of the participants. In previous reports, decreased LVEF was more common, as it was present, during the acute illness, in 34.2–40% of children with MIS-C [14, 16, 39]. In line with the findings of previous studies, that described a rapid improvement of LVEF, returning to normal by the first days to weeks from admission, all the participants of our cohort had preserved LVEF on follow-up [12, 13].

In parallel, during the acute phase, in a subset of MIS-C patients we observed an impairment of LV-GLS. Specifically, we found that during the acute phase, LV-GLS was impaired in 42.9% of the patients, while LVEF was normal in all but one of these patients. In MIS-C, LV dysfunction, identified by LV-GLS, during the acute illness, has been reported in other studies, where the rate of GLS impairment was estimated at 44–65% [23, 40]. Study data from mid-term echocardiographic follow-up evaluation (approximately at 2 months), report that while LV-GLS was improved in most patients, there was a persistence of GLS abnormalities in 21–24% of MIS-C patients [10, 23]. Upon long-term follow-up (range: 5–18 months), although all children had normal LVEF, in one patient we detected persistence of the GLS impairment. Notably, in our group of patients, while there was a trend for improvement in LV-GLS measurements, it was not statistically significant, probably due to the small sample size. Our findings are consistent with those reported by other studies, that observed a normalization of the LV-GLS in the majority of MIS-C patients at a six-month follow-up, where the rates of participants with residual GLS impairment ranged from 0 to 13% [11, 12, 18].

Abnormal strain measurements with preserved LVEF, during the acute phase of MIS-C and in different follow-up periods, have been also described in previous studies [4, 17, 23, 41]. The above findings are suggestive of subclinical myocardial injury, that can be recognized with STE with LV-GLS analysis, but not with conventional echocardiographic parameters like the LVEF [11]. Indeed, the LVEF relies on volume overload and does not take into account ultrastructural alterations that may occur at a myocardial level and may lead to LV systolic dysfunction [20, 21]. In contrast, STE parameters, like GLS, provide extensive information on the active deformation of the LV, permitting the early identification of cardiac dysfunction [20, 42].

In MIS-C, the underlying pathophysiological mechanisms of cardiac dysfunction have not been yet fully elucidated [43]. A possible pathophysiological explanation of the cardiac dysfunction in MIS-C is myocardial inflammation in the setting of a post-viral immunological reaction and systematic hyperinflammation [13, 43]. Given that MIS-C is a rare and relatively newly identified disease, histologic and imaging findings that could allow a better understanding of the cardiac involvement that is described in the syndrome, are still lacking [13, 15]. In this context, advanced echocardiographic techniques, like STE with GLS analysis, may help us to gain an in-depth understanding of the pathophysiology of ventricular dysfunction in patients with MIS-C [43].

Notably, in MIS-C, GLS impairment has been associated with a higher risk of inotropic support, ICU admission, or mechanical ventilation [44]. Therefore, GLS imaging is an indicator of disease severity and could be applied at admission, for the risk stratification of patients, regarding the need of early therapeutic management and ICU admission [10]. GLS can also help to identify which patients are at a greater risk for developing persistent myocardial dysfunction after the acute illness and therefore may benefit from a long-term cardiac follow-up [13, 19].

In addition, since diastolic dysfunction is a finding in MIS-C, diastolic parameters with the use of conventional echocardiography were also evaluated in our study [33, 45]. In the present cohort, at admission and at follow-up, none of the children was diagnosed with diastolic dysfunction. However, no definite conclusions can be made, as diastolic function was evaluated in a very small number of patients, especially at admission.

Most of the MIS-C patients that participated in our study, and were evaluated for valvular dysfunction, were diagnosed with mild mitral and/or tricuspid regurgitation in the acute phase. Valvular dysfunction has been described in MIS-C with an estimated rate of 24–48% [46]. However, we cannot disregard the fact that some of these children may have had preexisting, unrelated to MIS-C, valvular regurgitation, that was undiagnosed. Indeed, it has been reported that in healthy children with no evidence of heart disease, tricuspid and less frequently mitral valve regurgitation can be present with variable severity [47]. Regarding specifically the tricuspid valve, all the children had mild tricuspid regurgitation according to the 2012 European Society of Cardiology Criteria for grading tricuspid regurgitation and no structural abnormalities of the valve were detected, but still we cannot draw definite conclusions as to whether the tricuspid regurgitation was preexisting or not [48].

In the current study, in consistency with previous reported data, coronary arteries were spared in most of the participants [4]. Only one child had echocardiographic findings of coronary artery dilation that resolved on follow-up. CAA in MIS-C are described in published literature and usually are mild and transient [8]. However, the incidence of CAA in MIS-C varies among different studies and it is estimated to be at 6–24% [8, 43]. These discrepancies could be a result of the overlap of MIS-C with KD, a delay in the diagnosis and treatment, demographic disparities of the study populations, the circulating SARS-CoV-2 variant, the use of different MIS-C diagnostic criteria and variabilities in the quality of the applied imaging techniques [12, 15]. The underlying mechanism of CAA formation may also differ between KD and MIS-C [15, 16]. In KD, CAA are attributed to a disruption in the integrity of the wall of the coronary artery [15, 16]. In MIS-C, while the mechanism of coronary dilation is not fully elucidated, CAA could be a result of the vasodilation caused by fever and inflammation [15, 16, 49].

The major limitations of the present study are its single-center study design and the limited number of MIS-C patients. Also, other limitations are that GLS was performed only in a subset of the participants and the differences in the timing of the follow-up examination due to the different clinical demands of each patient. Furthermore, the evaluation of diastolic function and the STE-GLS analysis were performed only in a subset of the participants. Regarding the limitations of the echocardiographic methods used in the study, M-mode echocardiography is limited by angle-dependency, while the main limitation of STE is intervendor variability [50, 51]. Moreover, interobserver and intraobserver variability were not assessed in our study. Additionally, although, GLS is a very sensitive indicator of ventricular injury and is the most commonly used STE parameter in clinical practice, STE analysis was limited only in the evaluation of systolic function with longitudinal cardiac deformation assessment (GLS) and global circumferential and radial strain were not assessed [50]. Finally, we did not perform GLS with Cardiac Magnetic Resonance (CMR) imaging for comparison with the 2D-Ultrasound GLS results.

However, to the best of our knowledge, this is one of the very few prospective studies that provides echocardiographic longitudinal data with the use of both conventional and advanced echocardiographic (STE with LV-GLS analysis) techniques at a long-term follow-up period (at a median of 6.9 months post the acute illness). Besides, our study provides data for patients that were diagnosed with MIS-C during the Alpha, Delta and Omicron predominance periods.

## Conclusion

During the acute phase and the follow-up, we observed that a subset of children had abnormal LV-GLS values, in the presence of normal LVEF, indicating subclinical myocardial injury. This persistence of subclinical myocardial dysfunction in a small subset of MIS-C patients emphasizes the need of a cardiological follow-up after discharge. Our findings suggest that STE-GLS is a valuable tool for the detection of subclinical myocardial injury in MIS-C.

## References

[1] Nunziata F, Salomone S, Catzola A, Poeta M, Pagano F, Punzi L, Lo Vecchio A, Guarino A, Bruzzese E (2023) Clinical presentation and severity of SARS-CoV-2 Infection compared to respiratory Syncytial Virus and other viral Respiratory Infections in children less than two years of age. Viruses 15 10.3390/v15030717 PMC10055850 36992426

[2] Waseem M, Shariff MA, Tay ET, Mortel D, Savadkar S, Lee H, Kondamudi N, Liang T (2022). Multisystem inflammatory syndrome in children. J Emerg Med.

[3] Weatherhead JE, Clark E, Vogel TP, Atmar RL, Kulkarni PA (2020). Inflammatory syndromes associated with SARS-CoV-2 Infection: dysregulation of the immune response across the age spectrum. J Clin Invest.

[4] Matsubara D, Kauffman HL, Wang Y, Calderon-Anyosa R, Nadaraj S, Elias MD, White TJ, Torowicz DL, Yubbu P, Giglia TM, Hogarty AN, Rossano JW, Quartermain MD, Banerjee A (2020). Echocardiographic findings in Pediatric Multisystem Inflammatory Syndrome Associated with COVID-19 in the United States. J Am Coll Cardiol.

[5] Sirico D, Basso A, Reffo E, Cavaliere A, Castaldi B, Sabatino J, Meneghel A, Martini G, Da Dalt L, Zulian F, Di Salvo G (2021) Early echocardiographic and cardiac MRI findings in Multisystem Inflammatory Syndrome in Children. J Clin Med 10 10.3390/jcm10153360 PMC8348478 34362141

[6] Alkan G, Sert A, Oz SKT, Emiroglu M, Yılmaz R (2021). Clinical features and outcome of MIS-C patients: an experience from Central Anatolia. Clin Rheumatol.

[7] Renson T, Forkert ND, Amador K, Miettunen P, Parsons SJ, Dhalla M, Johnson NA, Luca N, Schmeling H, Stevenson R, Twilt M, Hamiwka L, Benseler S (2023). Distinct phenotypes of multisystem inflammatory syndrome in children: a cohort study. Pediatr Rheumatol Online J.

[8] Mannarino S, Raso I, Garbin M, Ghidoni E, Corti C, Goletto S, Nespoli L, Santacesaria S, Zoia E, Camporesi A, Izzo F, Dilillo D, Fiori L, D’Auria E, Silvestri A, Dolci A, Calcaterra V, Zuccotti G (2022). Cardiac dysfunction in Multisystem Inflammatory Syndrome in Children: an Italian single-center study. Ital J Pediatr.

[9] He M, Leone DM, Frye R, Ferdman DJ, Shabanova V, Kosiv KA, Sugeng L, Faherty E, Karnik R (2022). Longitudinal Assessment of Global and Regional Left ventricular strain in patients with multisystem inflammatory syndrome in children (MIS-C). Pediatr Cardiol.

[10] Sanil Y, Misra A, Safa R, Blake JM, Eddine AC, Balakrishnan P, Garcia RU, Taylor R, Dentel JN, Ang J, Cashen K, Heidemann SM, Bauerfield C, Sethuraman U, Farooqi A, Aggarwal S, Singh G (2021). Echocardiographic Indicators Associated with adverse clinical course and Cardiac Sequelae in Multisystem Inflammatory Syndrome in Children with Coronavirus Disease 2019. J Am Soc Echocardiogr.

[11] Sirico D, Basso A, Sabatino J, Reffo E, Cavaliere A, Biffanti R, Cerutti A, Castaldi B, Zulian F, Da Dalt L, Di Salvo G (2022). Evolution of echocardiographic and cardiac magnetic resonance imaging abnormalities during follow-up in patients with multisystem inflammatory syndrome in children. Eur Heart J Cardiovasc Imaging.

[12] Garbin M, Raso I, Piersanti A, Gianolio L, De Silvestri A, Calcaterra V, Corti CG, Nespoli LF, Santacesaria S, Fini G, Dilillo D, Zuccotti G, Mannarino S (2022) Advanced echocardiographic analysis in medium-term Follow-Up of children with previous multisystem inflammatory syndrome. Child (Basel) 9 10.3390/children9060917 PMC9222005 35740854

[13] Kobayashi R, Dionne A, Ferraro A, Harrild D, Newburger J, VanderPluym C, Gauvreau K, Son MB, Lee P, Baker A, de Ferranti S, Friedman KG (2021). Detailed Assessment of Left ventricular function in Multisystem Inflammatory Syndrome in Children, using strain analysis. CJC Open.

[14] Ludwikowska KM, Moksud N, Tracewski P, Sokolski M, Szenborn L (2023) Cardiac involvement in patients with multisystem inflammatory syndrome in children (MIS-C) in Poland. Biomedicines 11 10.3390/biomedicines11051251 PMC10215748 37238922

[15] Friedman KG, Harrild DM, Newburger JW (2020). Cardiac Dysfunction in Multisystem Inflammatory Syndrome in children: a call to action. J Am Coll Cardiol.

[16] Campanello C, Mercuri C, Derchi M, Trocchio G, Consolaro A, Caorsi R, Ravelli A, Rimini A, Marasini M, Gattorno M (2022) Cardiovascular manifestations in Multisystem Inflammatory Syndrome in Children (MIS-C) Associated with COVID-19 according to Age. Child (Basel) 9 10.3390/children9050583 PMC9139768 35626760

[17] Minocha PK, Srinivasan R, Babb J, Singh RK, Phoon CKL, Better D, Bhatla P (2022). Strain in children with MIS-C and acute COVID-19. Ann Pediatr Cardiol.

[18] Kamińska H, Rożnowska-Wójtowicz A, Cacko A, Okarska-Napierała M, Kuchar E, Werner B (2023) Three-Dimensional Echocardiography and Global Longitudinal Strain in Follow-up after MIS-C. J Pediatr: 113516 10.1016/j.jpeds.2023.113516 37244577

[19] McAree D, Hauck A, Arzu J, Carr M, Acevedo J, Patel AB, Husain N (2022) Clinical Predictors of Subacute Myocardial Dysfunction in Multisystem Inflammatory Syndrome in Children (MIS-C) Associated with COVID-19. Pediatr Cardiol: 1–12 10.1007/s00246-022-03021-9 PMC9580417 36260103

[20] Tops LF, Delgado V, Marsan NA, Bax JJ (2017). Myocardial strain to detect subtle left ventricular systolic dysfunction. Eur J Heart Fail.

[21] Hegazy M, Ghaleb S, Das BB (2023) Diagnosis and management of Cancer Treatment-Related Cardiac Dysfunction and Heart Failure in children. Child (Basel) 10 10.3390/children10010149 PMC9856743 36670699

[22] Wang B, Yu Y, Zhang Y, Hao X, Zhao H, Yang S, Sun Q, Wang Y (2020). Speckle tracking echocardiography in the early detection and prediction of anthracycline cardiotoxicity in diffuse large B-cell Lymphoma treated with (R)-CHOP regimen. Echocardiography.

[23] Das N, Hill R, Trivedi M, Kenkre TS, Alsaied T, Feingold B, Harris TH, Christopher AB (2023). Longitudinal Assessment of Cardiac function following multisystem inflammatory syndrome in Children Associated with COVID-19. Pediatr Cardiol.

[24] CDC Health Alert Network. Multisystem in- flammatory syndrome in children (MIS-C) associ- ated with coronavirus disease 2019 (COVID-19)

[25] World Health Organization Scientific Brief. Multisystem inflammatory syndrome in children and adolescents with COVID-19.

[26] Nir A, Lindinger A, Rauh M, Bar-Oz B, Laer S, Schwachtgen L, Koch A, Falkenberg J, Mir TS (2009). NT-pro-B-type natriuretic peptide in infants and children: reference values based on combined data from four studies. Pediatr Cardiol.

[27] Chen Z, He J, Ma Q, Xiao M (2021). Association between C-Peptide level and subclinical myocardial Injury. Front Endocrinol (Lausanne).

[28] Lai WW, Geva T, Shirali GS, Frommelt PC, Humes RA, Brook MM, Pignatelli RH, Rychik J (2006). Guidelines and standards for performance of a pediatric echocardiogram: a report from the Task Force of the Pediatric Council of the American Society of Echocardiography. J Am Soc Echocardiogr.

[29] Tissot C, Singh Y, Sekarski N (2018). Echocardiographic evaluation of ventricular function-for the neonatologist and Pediatric Intensivist. Front Pediatr.

[30] McCrindle BW, Rowley AH, Newburger JW, Burns JC, Bolger AF, Gewitz M, Baker AL, Jackson MA, Takahashi M, Shah PB, Kobayashi T, Wu MH, Saji TT, Pahl E (2017). Diagnosis, treatment, and long-term management of Kawasaki Disease: A Scientific Statement for Health professionals from the American Heart Association. Circulation.

[31] Nagueh SF, Smiseth OA, Appleton CP, Byrd BF 3rd, Dokainish H, Edvardsen T, Flachskampf FA, Gillebert TC, Klein AL, Lancellotti P, Marino P, Oh JK, Popescu BA, Waggoner AD (2016) Recommendations for the evaluation of left ventricular diastolic function by Echocardiography: an update from the American Society of Echocardiography and the European Association of Cardiovascular Imaging. J Am Soc Echocardiogr 29:277–314 10.1016/j.echo.2016.01.011 27037982

[32] Ho CY, Solomon SD (2006). A clinician’s guide to tissue doppler imaging. Circulation.

[33] Capone CA, Misra N, Ganigara M, Epstein S, Rajan S, Acharya SS, Hayes DA, Kearney MB, Romano A, Friedman RA, Blaufox AD, Cooper R, Schleien C, Mitchell E (2021) Six Month follow-up of patients with multi-system inflammatory syndrome in children. Pediatrics 148 10.1542/peds.2021-050973 34326176

[34] Boston Children’s Hospital Heart Center Z score calculator

[35] Karagözlü S, Ramoğlu MG, Bayram Ö, Bakhtiyarzada J, Aydın A, Yılmaz MM, Murt B, Özkan E, İnceli HB, Gurbanov A, Şükriye Y, Demir B, Özdemir H, Çiftçi E, Kendirli T, Uçar T, Fitoz ÖS, Tutar E (2023) Cardiovascular manifestations and cardiac magnetic resonance follow-up of multisystem inflammatory syndrome in children (MIS-C). Cardiol Young :1–10 10.1017/S1047951123001348 37381829

[36] Reisner SA, Lysyansky P, Agmon Y, Mutlak D, Lessick J, Friedman Z (2004). Global longitudinal strain: a novel index of left ventricular systolic function. J Am Soc Echocardiogr.

[37] Levy PT, Machefsky A, Sanchez AA, Patel MD, Rogal S, Fowler S, Yaeger L, Hardi A, Holland MR, Hamvas A, Singh GK (2016). Reference ranges of left ventricular strain measures by two-Dimensional Speckle-Tracking Echocardiography in children: a systematic review and Meta-analysis. J Am Soc Echocardiogr.

[38] Hoste L, Van Paemel R, Haerynck F (2021). Multisystem inflammatory syndrome in children related to COVID-19: a systematic review. Eur J Pediatr.

[39] Feldstein LR, Tenforde MW, Friedman KG, Newhams M, Rose EB, Dapul H, Soma VL, Maddux AB, Mourani PM, Bowens C, Maamari M, Hall MW, Riggs BJ, Giuliano JS, Singh AR, Li S, Kong M, Schuster JE, McLaughlin GE, Schwartz SP, Walker TC, Loftis LL, Hobbs CV, Halasa NB, Doymaz S, Babbitt CJ, Hume JR, Gertz SJ, Irby K, Clouser KN, Cvijanovich NZ, Bradford TT, Smith LS, Heidemann SM, Zackai SP, Wellnitz K, Nofziger RA, Horwitz SM, Carroll RW, Rowan CM, Tarquinio KM, Mack EH, Fitzgerald JC, Coates BM, Jackson AM, Young CC, Son MBF, Patel MM, Newburger JW, Randolph AG (2021). Characteristics and outcomes of US children and adolescents with Multisystem Inflammatory Syndrome in Children (MIS-C) compared with severe Acute COVID-19. JAMA.

[40] Chang JC, Matsubara D, Morgan RW, Diorio C, Nadaraj S, Teachey DT, Bassiri H, Behrens EM, Banerjee A (2021). Skewed cytokine responses rather than the magnitude of the Cytokine Storm May Drive Cardiac Dysfunction in Multisystem Inflammatory Syndrome in Children. J Am Heart Assoc.

[41] Başar EZ, Usta E, Akgün G, Güngör HS, Sönmez HE, Babaoğlu K (2022). Is strain echocardiography a more sensitive indicator of myocardial involvement in patients with multisystem inflammatory syndrome in children (MIS-C) associated with SARS-CoV-2?. Cardiol Young.

[42] Tantawy AAG, Elsherif NHK, Habeeb NM, Hasan EM, Abdelhameed AE (2022). A two-dimensional speckle-tracking echocardiography for the diagnosis of early myocardial Disease in beta-thalassemia major patients. Ann Pediatr Cardiol.

[43] Sperotto F, Friedman KG, Son MBF, VanderPluym CJ, Newburger JW, Dionne A (2021). Cardiac manifestations in SARS-CoV-2-associated multisystem inflammatory syndrome in children: a comprehensive review and proposed clinical approach. Eur J Pediatr.

[44] Liu K, Yu J, Song G (2022). Global myocardial strain in Multisystem Inflammatory Syndrome in Children, Kawasaki Disease, and healthy children: A Network Meta-Analysis. Front Pediatr.

[45] Chakraborty A, Johnson JN, Spagnoli J, Amin N, McCoy M, Swaminathan N, Yohannan T, Philip R (2023). Long-Term Cardiovascular outcomes of Multisystem Inflammatory Syndrome in Children Associated with COVID-19 using an Institution based Algorithm. Pediatr Cardiol.

[46] Hejazi OI, Loke YH, Harahsheh AS (2021). Short-term Cardiovascular Complications of multi-system inflammatory syndrome in children (MIS-C) in adolescents and children. Curr Pediatr Rep.

[47] Colan SD, Sleeper LA (2020). Longitudinal variation in Presence and Severity of Cardiac Valve Regurgitation in Healthy Children. J Am Soc Echocardiogr.

[48] Arsalan M, Walther T, Smith RL 2nd, Grayburn PA (2017) Tricuspid regurgitation diagnosis and treatment. Eur Heart J 38:634–638 10.1093/eurheartj/ehv487 26358570

[49] Tong T, Yao X, Lin Z, Tao Y, Xu J, Xu X, Fang Z, Geng Z, Fu S, Wang W, Xie C, Zhang Y, Wang Y, Gong F (2022). Similarities and differences between MIS-C and KD: a systematic review and meta-analysis. Pediatr Rheumatol Online J.

[50] Iskander J, Kelada P, Rashad L, Massoud D, Afdal P, Abdelmassih AF (2022). Advanced Echocardiography techniques: the future stethoscope of systemic Diseases. Curr Probl Cardiol.

[51] Wang YH, Sun L, Li SW, Wang CF, Pan XF, Liu Y, Wu J, Guan XP, Zhang SL, Dun GL, Liu YL, Wang LY, Cui L, Liu Y, Lai YQ, Ding MY, Lu GL, Tan J, Yang XJ, Li YH, Zhang XT, Fan M, Yu JH, Zheng QJ, Ma CY, Ren WD (2023). Normal reference values for mitral annular plane systolic excursion by motion-mode and speckle tracking echocardiography: a prospective, multicentre, population-based study. Eur Heart J Cardiovasc Imaging.

